# Loading of green-synthesized cu nanoparticles on Ag complex containing 1,3,5-triazine Schiff base with enhanced antimicrobial activities

**DOI:** 10.1038/s41598-023-47358-4

**Published:** 2023-11-21

**Authors:** Elham Pormohammad, Pouya Ghamari kargar, Ghodsieh Bagherzade, Hamid Beyzaei

**Affiliations:** 1 Department of Chemistry, Faculty of Sciences, University of Birjand, Birjand, 97175-615 Iran; 2 Department of Chemistry, Faculty of Science, University of Zabol, Zabol, Iran

**Keywords:** Biochemistry, Plant sciences, Chemistry

## Abstract

The physicochemical properties of materials change significantly in nanometer dimensions. Therefore, several methods have been proposed for the synthesis of nanoparticles. Plant extracts and essential oils are applied as natural and economic resources to prepare nanomaterials especially metal nanoparticles. In this project, a green, simple and efficient method has been designed for the synthesis of Cu nanoparticles using *Purple cabbage* extract as a reducing and stabilizing agent. They were successfully loaded onto a new Ag complex containing 1,3,5-triazine Schiff base as ligand to form Cu@Ag-CPX nanocomposite. Phytochemical contents of extract were identified by standard qualitative analyses. The chemical structure of all synthesized compounds was characterized using spectral data. In FT-IR, coordination of C=N bond of Schiff base ligand to Ag^+^ ions shifted the absorption band from 1641 to 1632 cm^−1^. The UV–Vis spectrum of Cu@Ag-CPX nanocomposite shown the peak related to Cu nanoparticles in the region of around 251 nm. 5:7 molar ratio of Cu to Ag in Cu@Ag-CPX was determined using ICP-OES. The FESEM, TEM, and DLS techniques provided valuable insights into the morphology and size distribution of the nanocomposite, revealing the presence of rods and monodispersed particles with specific diameter ranges. These analyses of the nanocomposite displayed rods with diameters from 40 to 62 nm as well as monodispersed and uniform particles with average diameter of 45 nm, respectively. The presence of elements including carbon, nitrogen, oxygen, Cu and Ag was proved by EDX-EDS analysis. The XRD pattern of Cu@Ag-CPX shown the diffraction peaks of Cu and Ag particles at 2θ values of 10°–80°, and confirmed its crystalline nature. The inhibitory properties of the synthesized compounds were evaluated in vitro against four Gram-negative and two Gram-positive bacteria, as well as two fungal strains. The MIC, MBC and MFC values obtained from microdilution and streak plate sensitivity tests were ranged from 128 to 4096 µg ml^−1^. While Cu nanoparticles and Ag complexes were effective against some pathogens, they were not effective against all them. However, the growth of all tested microbial strains was inhibited by Cu@Ag-CPX nanocomposite, and makes it as a new promising antimicrobial agent. Modification of nanocomposite in terms of nanoparticle and complex can improve its blocking activities.

## Introduction

Due to increasing environmental problems, finding solutions to prevent damage to the earth has become an important matter^1–4^. For this purpose, economic and biocompatible approaches have become very significant in the field of material science and nanotechnology, which has greatly progressed in the development of efficient materials for the synthesis of a variety of compounds in macro, micro and nano scales under environmentally friendly processes^5–7^. Nanotechnology is one of the newest and most effective areas of investigation in material knowledge^8–10^, with a tangible impact on life sciences such as biomedicine and biotechnology^11,12^. The utilization of biologic materials such as plant extracts (including leafage, stems, fruit skin, seeds, etc.), bacteria, algae, and fungi is one of the most common methods to synthesize nanoparticles (NPs)^13–15^. These eco-friendly condition are more efficient and reliable than conventional methods due to their lower toxicity and better scalability^16–18^. Green methods may synthesize NPs with better specific size and morphology than traditional methods^19,20^. Major herbal extracts can be easily and inexpensively extracted^21^. Compositions in the plant extracts can act as regenerating and stabilizing agents during the biosynthesis of NPs. The reduction of metal ions using plant extracts has been known since the 1900s; however, the nature of the mitigating agents in the green synthesis of NPs is still not well understood. It is suggested that water-soluble plant metabolites such as phenolic compounds, flavonoids, alkaloids, terpenoids and coenzymes are responsible for the reduction of metal ions to metal NPs^22,23^. The synthesized metallic NPs have unique optical, catalytic, biological and electromagnetic properties and can be applied in different areas such as biochemistry, engineering, medicine, agriculture, electronics, biomedicine, and groundwater treatment^24–29^. Among metal NPs, Cu has attracted significant attention due to its non-toxicity, affluence, high optical sorption coefficient and low band energies, as well as use in reaction progress, semiconductor devices, solar energy conversion, gas measurement, antimicrobial materials, night light sources, emission devices and Li-ion electrodes^30–33^.

**Figure 1:**
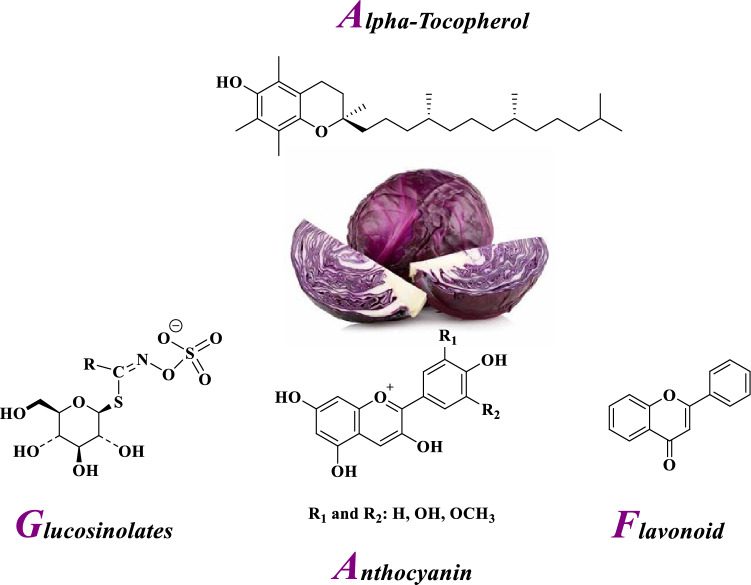
Various phytochemicals of *Purple cabbage.*

It is found that nanocomposites (NCs) containing metal NPs are potent antibacterial, antifungal and antioxidant agents^34,35^. Synergistic biological effects may be observed with them. The synthesis of NCs from renewable resources such as plant extracts offers advantages over artificial resources and provides a solution to environmental problems^36,37^. *Purple cabbage* (*Brassica oleracea var.capitata f. rubra*) is one of the plants that can be used for this purpose^38^. It is a herbaceous, two-year and dicotyledonous plant, native to the Mediterranean region and southwestern Europe, which grows all over the world^39^. It is useful in preventing heart diseases and stomach, prostate and breast cancers^40,41^. It as a nutrient-rich vegetable is available in fruit shops and grocery stores year round. It is believed that glucosinolates, polyphenols and anthocyanins in *purple cabbage* are responsible for its healing properties (Fig. 1)^42^.

It is considered as the best vegetable among *Brassica* species due to its highly efficient anti-radical system. Phenolic compounds are the main antioxidants in *Purple cabbage*, which inhibit or control food oxidation and play an important role in preventing oxidative stress^43–45^. Among polyphenolic compounds, anthocyanins in the plant have the ability to reduce metal ions to the corresponding nanoparticles^46^. The epidemic of drug-resistant infections poses a threat to global health, with reports estimating that 4.95 million people worldwide died of antibiotic-resistant bacteria in 2019^47^. In addition to preventive measures, new antimicrobial agents should be designed to control and fight these pathogens. Ag and Cu sources such as metal NPs and complexes are efficient antimicrobial agents that can be used alone or in combination with antibiotics and antifungals to treat drug-resistant pathogens^48,49^. Ag^+^ and Cu^2+^ ions released from these sources can stop microbial growth via several different mechanisms^50,51^.

To diverse library of antimicrobial agents, a new nanocomposite consisting of Cu NPs loaded on a Ag-Schiff base complex (Cu@Ag-CPX) was synthesized. Cu NPs themselves were prepared in the presence of *purple cabbage* extract. Inhibitory potentials of NPs, ligand, complex and nanocomposite were studied against various bacterial and fungal pathogens.

## Experimental

Supporting information including chemicals, solvents, microbial strains, instruments and spectral data are available in the Supplementary file.

### Preparation of plant extract

In February 2022, fresh *purple cabbage* was purchased from Ghafari market at the Birjand city, South Khorasan, Iran. The Purchased samples was for academic purposes, with permission from the university, comply with relevant institutional, national, and international guidelines and legislation. The sampling comply according with the IUCN Policy Statement on Research Involving Species at Risk of Extinction and the Convention on the Trade in Endangered Species of Wild Fauna and Flora. The waste and rotten leaves of freshly collected *purple cabbage* were removed and the fresh leaves were washed with distilled water (4 × 150 ml). The *cabbage* was then dried for two days under sterile conditions. Extraction of secondary metabolites was performed using the soaking method. To conduct the experiment, 70 g of chopped *purple cabbage* leaves were added to 250 ml of MeOH, EtOH or H_2_O and stirred at 25 ℃ for 72 h. The resulting extracts were served for phytochemical tests and synthesis of Cu NPs^52^.

### Identification of phytochemical compounds of plant extracts

According to the quantitative identification of the phytochemical compounds of purple cabbage extract in previous studies^53–56^, in this study, the qualitative identification of the phytochemical compounds of the plant extract using standard techniques to identify the different chemical groups present in the extracts^57–59^.

*Identification of Flavonoid*

The identification of flavonoids was done by adding 1 ml of concentrated sulfuric acid and 0.5 g of magnesium to 3 ml of each extract. The presence of flavonoids was indicated by the appearance of red or pink colors, which vanished after 3 min.

*Identification of Tannin*

To identify tannins, 3 ml of ethanolic, methanolic, and aqueous extracts of *purple cabbage* were added to separate test tubes, and 2–3 drops of 1% ferric chloride were added to each. Green to blue-green color (Kachi tannins) or blue-black color (Galley tannins) indicated the presence of tannins.

*Identification of Alkaloid*

Two procedures were used to identify alkaloids. First, 3 ml of each extract was heated, and then 3 ml of 2% HCl acid solution was added to each. The presence of alkaloidal base was indicated by the appearance of yellow-white precipitate after adding 1–2 drops of Wagner's reagent. Second, a few drops of Dragendorff's reagent were added, and the presence of a brown precipitate indicated the presence of alkaloids.

*Identification of Glucoside*

To identify glucoside, 20 drops of Fehling's solution were added to 3 ml of each extract prepared in a test tube. The appearance of a red precipitate at the end of the tube indicated the presence of glucosides.

*Identification of Protein*

Protein identification was done by adding 2 drops of concentrated nitric acid to the extract, and the presence of protein was indicated by the appearance of a red color. The amino acid tyrosine must be present in the protein.

*Identification of Amino acid*

The presence of amino acid was determined using the ninhydrin test. For this purpose, 2 ml of ninhydrin reagent was added to 3 ml of each extract and was boiled for 15 min. The formation of a purple color indicated the presence of amino acids.

### Biosynthesis of Cu NPs using *purple cabbage* extract

8 ml of methanolic *purple cabbage* extract was added to 40 ml of a 0.1 M aqueous solution of Cu(OAc)_2_ and subjected to ultrasound for 2 h. The resulting mixture was centrifuged and a black precipitate was obtained. The obtained powder was then dried in an oven at 65 °C for 6 h.

### Synthesis of schiff base (SM)

2,2′,2″-((1Z,1′Z,1″Z)-((1,3,5-triazine-2,4,6-triyl)tris(azaneylylidene))tris(methaneylylidene)) triphenol Schiff base (SM) was prepared in terms of the previously described method^60^. 0.5 g of melamine was added to 150 ml of DMF and heated to 120 °C for 2 h to dissolve it and obtain a clear solution. Then, 1.3 ml of salicylaldehyde was added dropwise to it. The solution gradually turned pale yellow, and the mixture was refluxed at 120 °C for 4 h. After the reaction mixture was cooled to 25 °C, it was slowly added to a beaker containing 300 mL of toluene, which resulted in the formation of a pale-yellow solid. The prepared solid was washed with a mixture of equal volume of toluene and methanol (3 × 25 ml). Finally, the resulting yellow powder was dried in an oven at 110 °C for 2 h.

### Synthesis of Ag complex (Ag-CPX)

A solution of AgNO_3_ in ethanol (0.5 g: 5 ml) was added dropwise to a container containing 0.2 g of SM and 10 ml of ethanol while sonicating. After sonication for 15 min, the reaction mixture was stirred at room temperature for 2 h. Finally, the resulting black precipitate was collected by centrifugation (5000 rpm), washed with ethanol, and dried at room temperature for 24 h.

### Synthesis of Cu@Ag-CPX nanocomposite

An ethanolic solution (5 ml) of synthesized Cu NPs (0.2 g) was added dropwise under ultrasound for 15 min to a reaction vessel containing 0.2 g of Ag-CPX complex and 10 ml of EtOH. Then the reaction mixture was stirred for 60 min at room temperature. The obtained black precipitate was centrifuged (5000 rpm) for 10 min. Finally, the black solid was washed with ethanol (3 × 10 ml) and dried at room temperature for overnight.

### Antimicrobial susceptibility tests

Broth microdilution and streak plate methods according M07-A9, M27-A2, M38-A2 and M26-A CLSI (Clinical and Laboratory Standards Institute) protocols were applied to determine the minimum inhibitory concentration (MIC), minimum bactericidal concentration (MBC) and minimum fungicidal concentration (MFC) values^57,62^. A suspension of bacterial and fungal strains were prepared at concentrations of 5 × 10^5^ and 2.5 × 10^3^ CFU/ml, respectively. All synthesized compounds were dissolved in DMSO at an initial concentration of 40,960 μg/ml and two-fold serially diluted in a 96-well microtiter. 100 μl of the microbial suspensions and 80 μl of broth culture media (Mueller–Hinton for bacteria and Sabouraud dextrose for fungi) were added to each well of the microplate containing 20 μl of diluted solution of compounds to obtain final concentrations 4096, 2048, 1024, 512, 256, 128, 64, 32, 16, 8, 4, and 2 μg/ml. The microplates were incubated at 37 °C for 24 h. Finally, the turbidity of the cultures was examined using plate-reading ELISA reader ELX800 (BioTek Instruments) and the lowest concentration at which the culture was transparent was reported as MIC. Also, to determine the MBC and MFC, 10 μg of the content of each clear well were transferred to Mueller–Hinton Agar and Sabouraud dextrose agar media, and incubated at 37 °C for 24 h. The concentration at which all microorganisms were eliminated was regarded as MBC or MFC. The results were presented as the average of three independent experiments. Ampicillin antibiotic and fluconazole antifungal were utilized as positive controls for the bacteria and fungi, respectively.

## Results and discussion

**Scheme 1:**
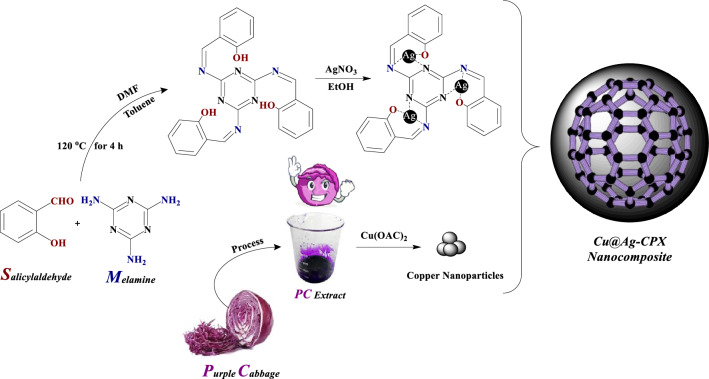
Schematic synthesis of Cu@Ag-CPX nanocomposite.

The current study describes the multi-step synthesis of Cu@Ag-CPX nanocomposite. In the first step, Cu NPs were obtained by a fast, environmentally friendly and biological process using *purple cabbage* extract as reducing agent at ambient temperature. Next, a Schiff base containing C=N bonds was formed via condensation of melamine with salicylaldehyde. Coordination of this ligand with Ag^+^ ions produced a Ag complex in the subsequent step. Finally, Cu NPs were loaded onto Ag complex to gain the Cu@Ag-CPX nanocomposite (Scheme 1).

**Scheme 2:**
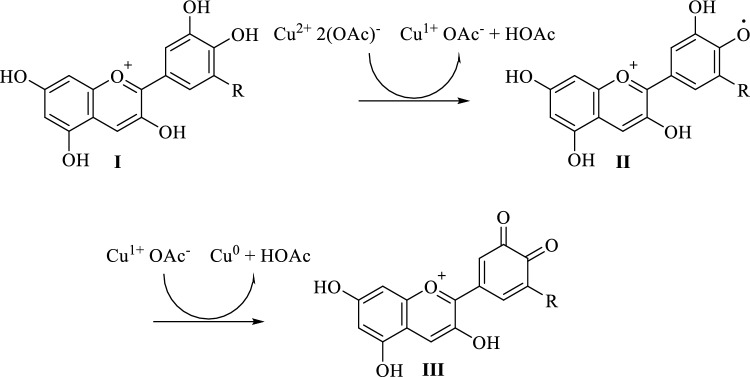
Possible mechanism of the formation of Cu NPs in the presence of *purple cabbage* extract.

Phenolic compounds, especially anthocyanins, in *purple cabbage* extract probably act as reducing agents. Scheme 2 illustrates a proposed mechanism for the formation of Cu NPs in the presence of anthocyanins based on interaction of caffeic acid in rice husk extract with Ag^+^ ions^63^. First, electrons released from anthocyanin **I** reduces Cu^2+^ to Cu^1+^. Further reduction occurs by electron absorption from intermediate **II** to convert Cu^1+^ to Cu^0^. Intermediate **II** itself is oxidized to *o*-quinone **III** during this process.

**Figure 2:**
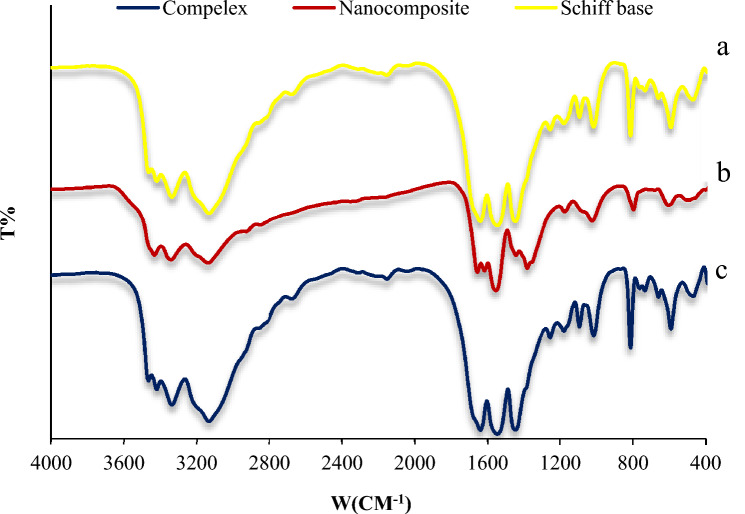
FT-IR spectra of Schiff base (**a**), Cu@Ag-CPX nanocomposite (**b**) and Ag-CPX complex (**c**).

The Cu@Ag-CPX nanocomposite was characterized using various techniques including Fourier transform infrared (FT-IR), field emission scanning electron microscopy (FESEM), transmission electron microscopy (TEM), X-ray diffraction (XRD), energy-dispersive X-ray spectroscopy (EDX-EDS) and ultraviolet–visible (UV–Vis). The FT-IR spectrum of the Schiff base (Fig. 2a) shows stretching vibrations of the O–H group at 3337 cm^−1^, stretching C–H bond of the aliphatic compounds and the aromatic ring at about 3313–2950 cm^−1^, and stretching vibrations of C=N (melamine) and C=C (aromatic ring) at 1641 cm^−1^ and 1448 cm^−1^, respectively.^64,65^ Absorption of imine bonds with metals will reduce the wavelength^66–68^, in this spectrum, C=N bonds absorption (1641 cm^−1^) shifted to lower wavenumbers (1632 cm^−1^) as a result of the coordination of Ag^+^ ions to Schiff base (SM) (Fig. 2c). Intensity and position of some peaks in FT-IR spectrum of Cu@Ag-CPX nanocomposite compared to Ag-CPX have changed due to the presence of Cu NPs (Fig. 2b). Peak at 604 cm^−1^ corresponds to stretching Cu NPs in nanocomposite^69^.

**Figure 3:**
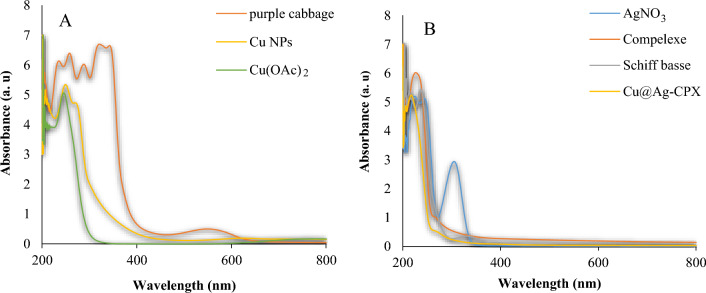
UV–Vis spectra of (**A**) Cu NP, *Purple cabbage*, Cu(OAc)_2_ and (**B**) Cu@Ag-CPX Nanocomposite, Schiff Bass, Ag-CPX complex, AgNO_3_.

Figure 3 displays the UV–Vis spectra of *purple cabbage* extract, Cu(OAc)_2_, Cu NPs, Cu@Ag-CPX nanocomposite, Ag-CPX complex, ligand SB and AgNO_3_. The results showed that the maximum absorption in the region of 251 nm belongs to Cu NPs which is in complete agreement with previous reports (Fig. 3A), indicating the reduction of Cu^2+^ ions using *purple cabbage* extract. The peak in the region of 245 nm corresponds to Cu(OAc)_2_, while the specific peaks in the range of 200–350 nm are related to phenolic compounds in *purple cabbage* extract (Fig. 3A)^70^. The surface plasmon resonance (SPR) bands in the range of 250–320 nm showed the presence of Ag particles (Fig. 3B)^71^. The peak near 245 nm corresponds to imine bonds of ligand SB. The significant peaks of AgNO_3_ and ligand SB are seen in Ag-CPX complex. The peak related to Cu NPs is also observed around 250 nm in the spectrum of Cu@Ag-CPX nanocomposite (Fig. 3B)^72^.

**Figure 4:**
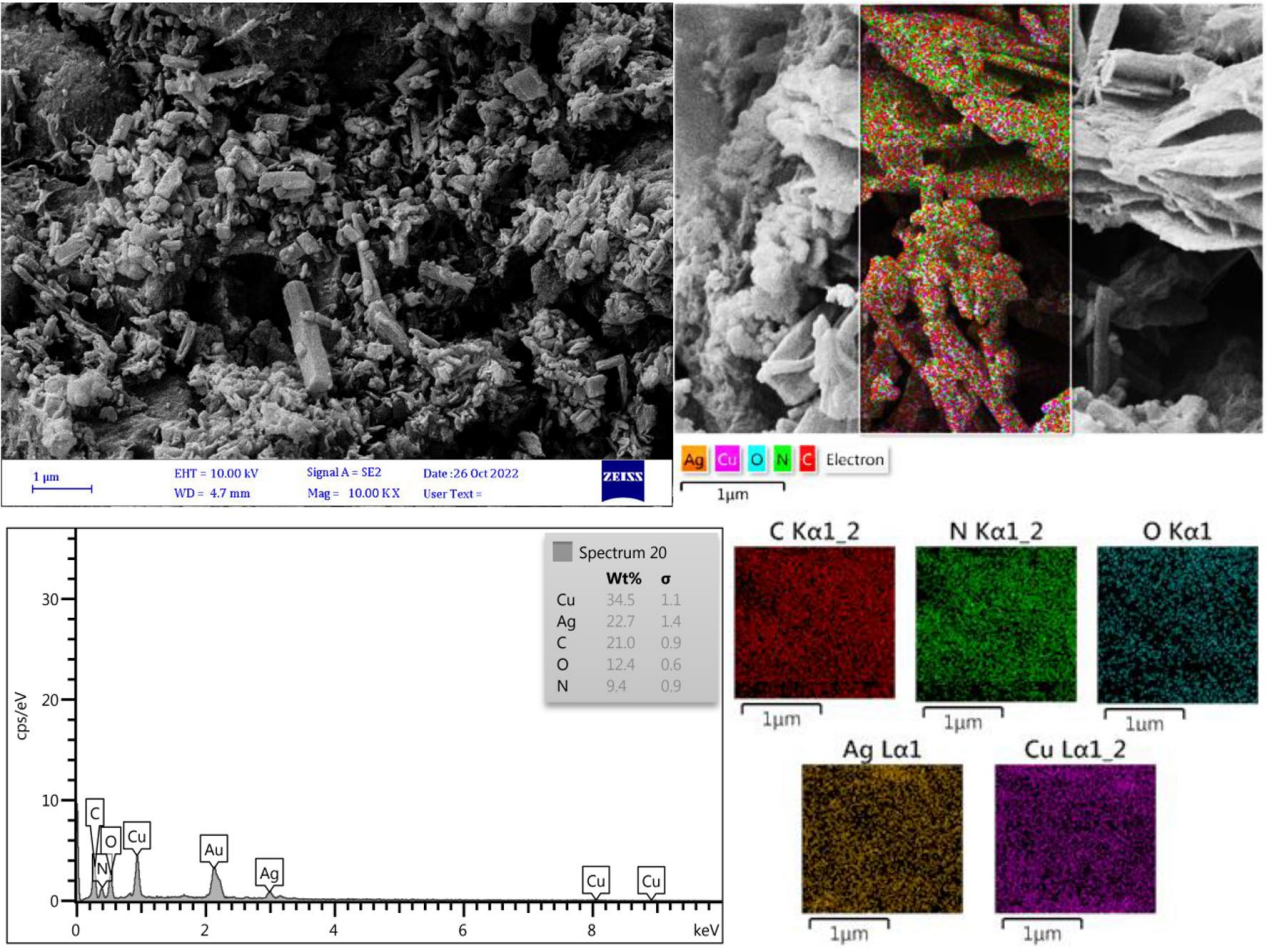
FESEM and EDX-EDS images of Cu@Ag-CPX nanocomposite.

The values of 1.72 × 10^–3^ and 1.26 × 10^–3^ mol g^−1^ were determined using inductively coupled plasma optical emission spectroscopy (ICP-OES) for Cu and Ag in the Cu@Ag-CPX nanocomposite, respectively. It shows an approximate molar ratio of 5:7 of Cu to Ag in the synthesized nanocomposite. Figure 4 displays the FESEM micrograph of the Cu@Ag-CPX nanocomposite rods with diameters between 40 to 62 nm. EDX-EDS analysis was also performed to identify the constituent elements of the synthesized nanocomposite, which included carbon, nitrogen, oxygen, Cu and Ag.

**Figure 5:**
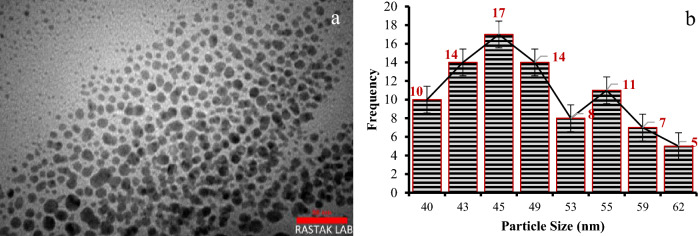
(**a**) TEM image and (**b**) DLS of Cu@Ag-CPX nanocomposite.

The particle size distribution of the Cu@Ag-CPX nanocomposite was determined via TEM and DLS analysis. The TEM image displayed monodispersed and uniformly sized particles within the nanocomposite. The TEM analysis provided a detailed view of the individual particles, showcasing their uniformity and distribution within the nanocomposite (Fig. 5a). Furthermore, DLS analysis was conducted to determine the hydrodynamic diameter of the nanocomposite particles in solution. The results indicated that the particles maintained their uniformity and exhibited an average hydrodynamic diameter of 45 nm (Fig. 5b) while were smaller than spherical Cu NPs prepared using *dicliptera roxburghiana* extract with the estimated size of 58 nm.^69^.

**Figure 6:**
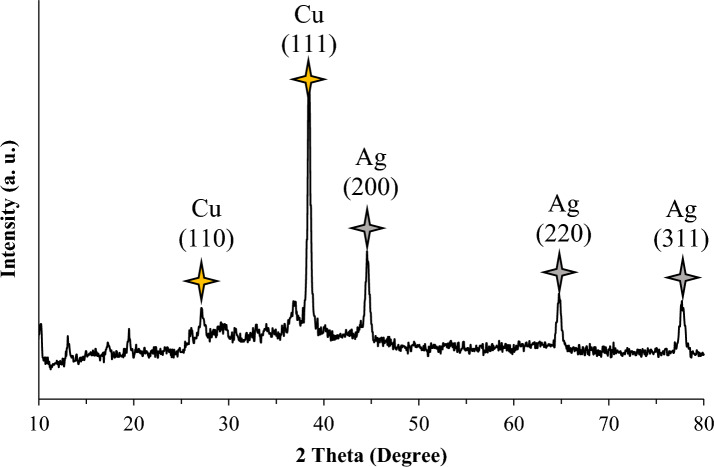
XRD of Cu@Ag-CPX nanocomposite.

Figure 6 shows the XRD pattern of Cu@Ag-CPX. 2θ values of 10–80° were obtained for Cu and Ag in the nanocomposite. The pattern revealed the diffraction peaks of Cu NPs at 2θ values of 38.5° and 29.15°, with 111 and 110 lattice planes, respectively^73^. Additionally, Ag particles were observed at 44.75, 64.85 and 77.95° with grid planes of 200, 220, and 311, respectively, which correspond to the central card number corresponding to the HCP (hexagonal close packed) cubic structure (04–0783)^70^. The XRD confirmed crystalline nature of the Cu@Ag-CPX NCs.

### Phytochemical analysis

Table 1 summarizes the phytochemical properties of *purple cabbage* extracts, indicating the presence of biologically active compounds in this plant. Tannins, flavonoids, protein, and amino acids were detected in *purple cabbage*, while alkaloids and sugars were not present in it, as shown in Table 2.

**Table 1:** Phytochemical test of methanolic, ethanolic and aqueous extracts of the *purple cabbage.*

Family of compounds	Alkaloids	Wagner	Dragendorff	Tannins	Flavonoids	Sugars	Amino acid	Protein
Ethanol extract	−	−	−	+ + +	+ + +	−	+	+
Methanol extract	−	−	−	+ + +	+ + +	−	+	+
Aqueous extract	−	−	−	+	+	−	+ +	+ +

(−): Negative test; ( +): Weak positive test; (+ +): Positive test; (+ + +): Strongly positive test.

**Table 2:** Active compounds in *purple cabbage* extract.

Family of compounds	Structure	Results
Tannins		
Flavonoids		
Amino acid		
Protein		

Table 2 displays the phytochemical analysis images of the ethanolic, methanolic, and aqueous extracts of *purple cabbage*, which revealed the abundance of tannin and flavonoid compounds in the plant. Tannins are secondary metabolites found in various plants and responsible for their antimicrobial properties. They also possess analgesic and anti-inflammatory activities and have astringent properties, which promote faster healing of ulcers and swollen mucous membranes. Phenolic compounds are a ubiquitous group of plant metabolites that have various biological properties such as anti-aging, anti-cancer, anti-inflammatory, anti-arteriosclerosis, and protective effects on the heart and blood vessels. They can also inhibit angiogenesis and cell proliferation activities. Flavonoids are hydroxylated phenolic substances synthesized by plants in response to microbial infections. Biomolecules especially anthocyanins in the *purple cabbage* extract can act as reducing and stabilizing agents to reduce metal ions to nano-sized materials.

### Antimicrobial test

**Figure 7:**
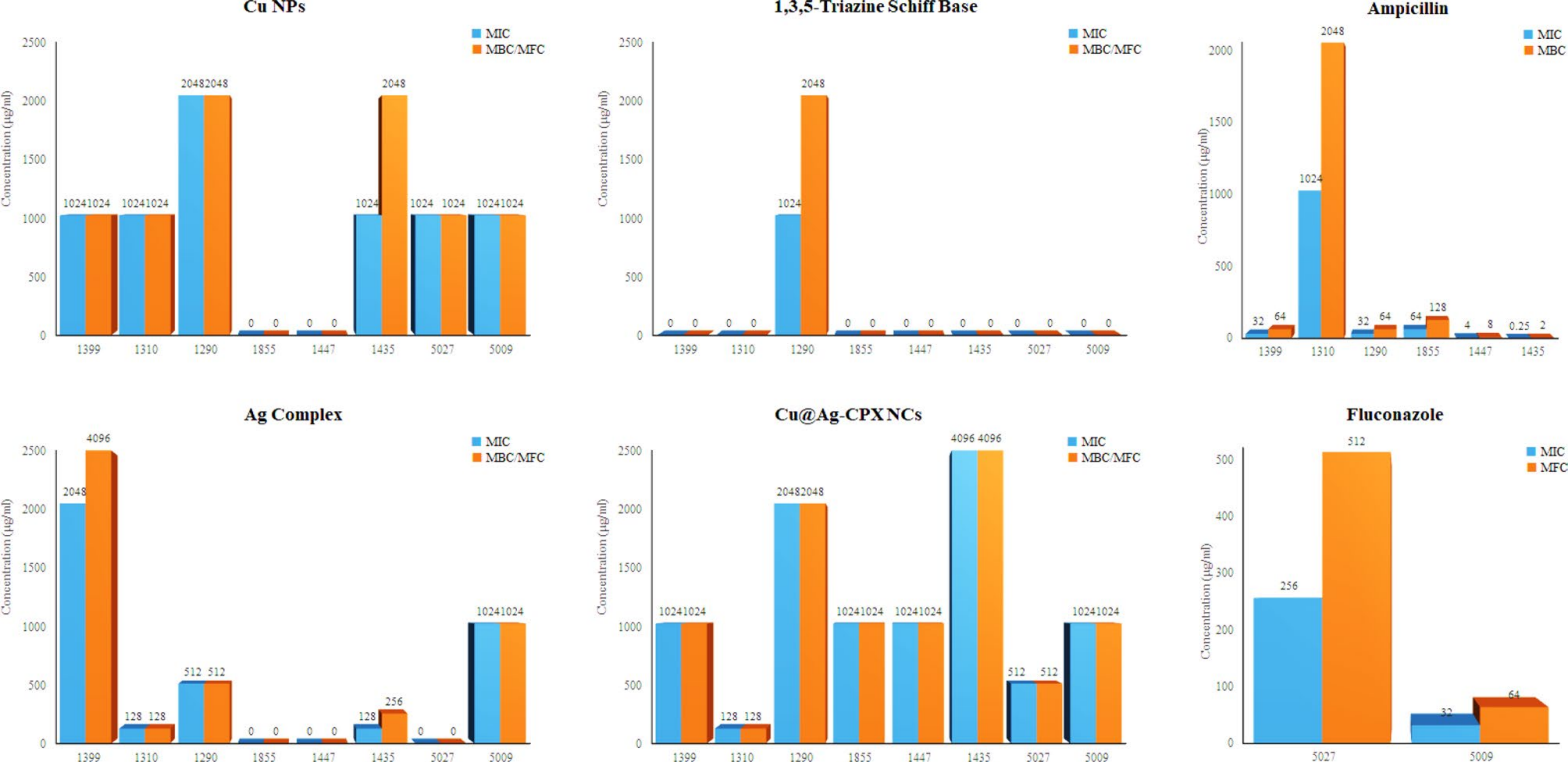
MIC, MBC and MBC values of compounds and drugs; a zero value shows no inhibitory activity in the highest concentration (4096 μg ml^−1^); 1399: *E. coli*, 1310: *P. aeruginosa*, 1290: *K. pneumoniae*, 1855: *A. baumannii*, 1447: *S. pyogenes*, 1435: *S. epidermidis*, 5027: *C. albicans*, 5009: *A. fumigatus.*

The synthesized compounds were studied for their antimicrobial potentials on some pathogenic bacteria and fungi. The results were compared with inhibitory activities of ampicillin and fluconazole (Fig. 7). Cu@Ag-CPX nanocomposite could inhibit the growth of all tested microorganisms. *A. baumannii* and *S. pyogenes* strains were stopped only by it. Schiff base was only effective on *K. pneumoniae*. Synergistic antimicrobial effects against *A. baumannii*, *S. pyogenes* and *C. albicans* were observed by Cu@Ag-CPX in comparison with Cu NPs and Ag-CPX. These results confirm that the release of Ag^+^ and Cu^2+^ ions either alone or together in nanocomposite occurs to inhibit bacteria and fungi. Antibacterial effects of nanocomponds based on Ag^+^ ions bound to carriers such as titan yellow and albumin were investigated against *E. coli* and *S. aureus* strains.^74^ The MIC and MBC values were reported in the range of 0.019–1.12 mM. Polymyxin B peptide is a naturally occurring antibiotic that can synergistically enhance antimicrobial activity of both Ag and Cu NPs.^75,76^ Inhibitory properties of multi-concentration of Cu:Ag bimetallic NPs were studied by Mureed et al*.* against antibiotic-resistant bacteria.^77^ It was found that increasing the concentration of Ag NPs improves antibacterial effects especially on Gram-positive strains.

## Conclusion

This study presents an environmentally friendly and effective method for synthesizing Cu@Ag-CPX nanocomposite using *purple cabbage* extract. It is predicted that polyphenolic compounds in *purple cabbage* extract were responsible to reduce Cu^2+^ ions to Cu^0^ NPs. The morphology of biosynthesized nanocomposite was characterized using various techniques. The antibacterial and antifungal potentials of all compounds were also evaluated against various pathogenic bacteria and fungi. Synergistic effects were observed with Cu NPs loaded on Ag-CPX complex against some pathogens. It is concluded that both Ag and Cu ions play a vital role in the mechanism of antimicrobial action of Cu@Ag-CPX nanocomposite. This nanocomposite may also serve as an efficient inhibitory agent and disinfectant against other microorganisms such as parasites and viruses. In addition, the synthesized Cu NPs have high surface area and can be used for different purposes.

### Supplementary Information

Supplementary Information.

## Data Availability

All data generated or analyzed during this study are included in this published article (and its Supplementary Information files).
