# Supplementary material for: Loading of green-synthesized cu nanoparticles on Ag complex containing 1,3,5-triazine Schiff base with enhanced antimicrobial activities
Source: Sci Rep. 2023 Nov 21;13:20421. doi: 10.1038/s41598-023-47358-4 (PMC10663565; doi:10.1038/s41598-023-47358-4)
Supplement: Supplementary file 1 — Supplementary Information. [file 41598_2023_47358_MOESM1_ESM.pdf]

Loading of Green-Synthesized Cu Nanoparticles on Ag Complex Containing 1,3,5-Triazine Schiff Base with Enhanced Antimicrobial activities

Elham Pormohammad,[a] Pouya Ghamari kargar,[a] Ghodsieh Bagherzade,[a]* Hamid Beyzaei[b]*

[a] *Department of Chemistry, Faculty of Sciences, University of Birjand, Birjand, 97175-615, Iran.*

[b] *Department of Chemistry, Faculty of Science, University of Zabol, Zabol, Iran.*

**Corresponding author:* Bagherzadeh@birjand.ac.ir; Hbeyzaei@uoz.ac.ir

Materials and Method

All reagents, solvents and drugs including copper (II) acetate monohydrate, silver nitrate, toluene, DMF, salicylic aldehyde, ampicillin, fluconazole and α-tocopherol were purchased from company were purchased from Merck and Sigma-Aldrich companies, and used without further purification. FT-IR spectra were obtained using a JASCO FT/IR 4600 spectrophotometer using KBr pellet. The reaction progress and the purity of compounds were monitored using TLC analytical silica gel plates (Merck 60 F250). Field emission scanning electron microscopy (FE-SEM) images were obtained on a ZEISS Sigma 300. EDX spectroscopy was performed using a field emission scanning electron microscope (JEOL 7600F), transmission electron microscopy (TEM) was performed on a Philips EM208 microscope operated at 100 kV. ICP experiments were accomplished using a Varian Vista Pro CCD simultaneous ICP-OES instrument. The powder X-ray diffraction (XRD) was performed with a Philips PW1730. The antimicrobial activity of all synthesized compounds was investigated against 2 Gram-positive and 4 Gram-negative pathogenic bacteria including Staphylococcus epidermidis (PTCC 1435), Streptococcus pyogenes (PTCC 1447), Escherichia coli (PTCC 1399), Pseudomonas aeruginosa (PTCC 1310), Acinetobacter baumannii (PTCC 1855) and Klebsiella pneumoniae (PTCC 1290) and 2 fungi including Candida albicans (PTCC 5027) and Aspergillus fumigatus (PTCC 5009) from the Persian Type Culture Collection (PTCC), Karaj, Iran. Minimum inhibitory concentration (MIC), minimum bactericidal concentration (MBC) and minimum fungicidal concentration (MFC) values were determined by microdilution and streak plate methods.[1,2] All derivatives were dissolved in DMSO to give

initial concentrations of 40960 μg.ml^{-1}. All microbial tests were repeated three times and the results were expressed as the average of three independent experiments.

References

1. Beyzaei, H. *et al.* Green multicomponent synthesis, antimicrobial and antioxidant evaluation of novel 5-amino-isoxazole-4-carbonitriles. *Chem. Cent. J.* **12**, 114 (2018).
2. Beyzaei, H., Moghaddam-Manesh, M., Aryan, R., Ghasemi, B. & Samzadeh-Kermani, A. Synthesis and in vitro antibacterial evaluation of 6-substituted 4-amino-pyrazolo[3,4-d]pyrimidines. *Chem. Pap.* **71**, 1685–1691 (2017).
